# MiR-4334-5p Facilitates Foot and Mouth Disease Virus Propagation by Suppressing Interferon Pathways via Direct Targeting ID1

**DOI:** 10.3390/genes11101136

**Published:** 2020-09-27

**Authors:** Yanxue Wang, Tingting Ren, Haotai Chen, Kailing Wang, Yongguang Zhang, Lei Liu, Yuefeng Sun

**Affiliations:** 1 State Key Laboratory of Veterinary Etiological Biology, National Foot and Mouth Diseases Reference Laboratory, Key Laboratory of Animal Virology of Ministry of Agriculture, Lanzhou Veterinary Research Institute, Chinese Academy of Agricultural Sciences, Lanzhou 730046, China; wangyanxue115@163.com (Y.W.); rentingting96@163.com (T.R.); chenhaotai@caas.cn (H.C.); 15214060592@163.com (K.W.); zhangyongguang@caas.cn (Y.Z.); 2 College of Veterinary Medicine, Gansu Agricultural University, Lanzhou 730070, China; 3 Jiangsu Co-Innovation Center for Prevention and Control of Important Animal Infectious Diseases and Zoonoses, Yangzhou University, Yangzhou 225009, China

**Keywords:** miR-4334-5p, ID1, FMDV, interferon

## Abstract

Emerging evidence indicates that the host microRNAs (miRNAs) are important intracellular regulators and play pivotal roles in intricate host-pathogen interaction networks. In our previous studies, ssc-microRNA-4334-5p (miR-4334-5p) was identified as a differentially expressed miRNA in microarray-based miRNAs profiling experiment, but whether miR-4334-5p regulates foot and mouth disease virus (FMDV) propagation is less understood. Here, we demonstrated that miR-4334-5p expression level was up-regulated shortly after FMDV infection, transfection of miR-4334-5p mimics promoted, while inhibitor transfection suppressed FMDV replication correspondingly. Further bioinformatic analysis and experimental study suggested ID1 was the direct target of miR-4334-5p, suppressing FMDV replication by regulating interferon (IFN) pathways. These findings shed light on microRNAs-ID1-interferon axis in regulating FMDV replication.

## 1. Introduction

Foot and mouth disease is an acute and highly contagious disease, caused by foot and mouth disease virus (FMDV), which mainly occurred in cloven-hoofed animals, such as cattle, sheep, goats and swine. The clinical symptoms in infected animals are usually shown as the vesicles formation in the mouth, nose, tongue, and skin between the claws of the feet [1]. FMDV is a member of the *aphthovirus* genus of the family *Picornaviridae*, and the genome RNA is ~8.5 kb with positive strand. There are seven distinct serotypes in FMDV (serotypes O, A, C, Asia1, and SAT1~3), serotypes O and A continuously pose a threat to the animal husbandry industry currently in China [2]. The polyprotein is translated by the viral RNA, and subsequently cleaved into four structural proteins (VP1, VP2, VP3 and VP4), and eight nonstructural proteins (leader protein (L^pro^), 2A, 2B, 2C, 3A, 3B, 3C^pro^ and 3D^pol^) [3]. Upon the viral pathogen-associated molecular patterns (PAMPs) recognized by pattern recognize receptors (PRRs), a signaling cascade stimulating the expression of type-I interferon (IFN) and proinflammatory cytokines is initiated, and the host protective immune response is followed; thus establishing a balance of the host against viral infection. The interaction mechanisms between host and pathogen are extremely intricate. According to the previous studies, L^pro^, 3C^pro^ and VP3 of FMDV repress the IFN-I pathway [4,5,6]. However, emerging evidence indicates that the microRNAs in the hosts participate in the comprehensive host-pathogen regulatory mechanisms [7]. The underlying mechanisms of host microRNAs affecting FMDV replication are still not well understood and need further investigation.

MicroRNA, a small non-coding RNA with a length of 18~22 nt, typically regulate post-transcription of gene expression in immune responses [2,8]. The primary transcript (pri-miRNA) is cleaved to generate precursor miRNA (pre-miRNA) under the participation of RNase III enzyme Drosha in nucleus. Subsequently, the pre-miRNA is cleaved into a duplex RNA of 18∼22 nt by Dicer in cytoplasm. MiRNAs are loaded with Argonaut proteins to form the miRNA Induced Silencing Complexes (miRISCs) and function in repressing gene expression [9,10]. MiRNAs mainly target the 3′ noncoding region of mRNA, but may also target the coding sequence [11,12]. The interaction between miRNAs and mRNAs are based on complementary binding, dominated by the “seed” region which spans the 5′-terminal 2~8 bases of the microRNA [7]. Particularly, it is confirmed that miRNAs play crucial roles in viral infections. For instance, it is reported that microRNA-155 represses dengue virus reproduction by facilitating heme oxygenase-1-mediated interferon responses [13]. MicroRNA-802 was reported to induce hepatitis B virus propagation by altering the expression of SMARCE1 [14]. In case of FMDV, miR-203a-5p was reported to reduce FMDV viral RNA by regulating Sam68 [15]. Furthermore, our lab reported FMDV replication is repressed by miRNA-1307 via destabilizing VP3 [2]. In contrast, microRNA-4331-5p accelerates FMDV reproduction by interfering interferon pathways [16].

In our previous studies, miR-4334-5p was identified as a differentially expressed miRNA in miRNAs profiling assay in porcine PK-15 after challenging with FMDV serotype O strain (O/BY/CHA/2010) for 2 h. There are few studies about whether and how miR-4334 influences virus infection. Here we showed that miR-4334-5p was directly up-regulated upon FMDV infection, and further transfection of miR-4334-5p mimics or inhibitor promotes or inhibits FMDV replication correspondingly. Subsequent studies revealed that miR-4334-5p induces FMDV replication by targeting Inhibitor of DNA binding proteins 1 (ID1) to regulate interferon (IFN) pathways. Inhibitor of DNA binding proteins 1 (ID1), mainly reported as a transcriptional regulator that control the timing of cell fate determination and differentiation in stem and progenitor cells, also function as a biomarker in various types of human tumors [17]. This is the first report that miR-4334-5p induces viral replication. These data revealed that the host miR-4334-5p may be a candidate for RNA interference related antiviral therapeutic strategies.

## 2. Materials and Methods

### 2.1. Cells and Reagents

PK-15 (Porcine Kidney cells) and BHK-21 (Baby Hamster Kidney cells) were obtained from ATCC, maintained with 10% fetal bovine serum (FBS, AusgeneX, QLD, Australia) in high glucose Dulbecco’s modified Eagle’s medium (DMEM, Gibco, Carlsbad, CA, USA) in 37 °C and 5% CO_2_ incubator. VP1 polyclonal antibody were produced in rabbit (gifted by Dr. Haixue Zheng, Lanzhou Veterinary Research Institute, Lanzhou, China), both ID1 and β-actin monoclonal antibodies were obtained from Santa Cruz Biotechnology. Goat anti-rabbit or anti-mouse IgG antibody conjugated to horseradish peroxidase was purchased from Bio-Rad (Hercules, CA, USA).

### 2.2. Virus and Viral Challenge Assays

FMDV serotype O strain O/BY/CHA/2010 was used for viral infection assays in this study, and offered by OIE/National Foot-and-Mouth Disease Reference Laboratory (Lanzhou, China). The propagation and titrations of FMDV were performed in BHK-21cells. All of the experiments related to viral-challenge were carried out in the Biosafety Level-3 (BSL-3) Laboratory of Lanzhou Veterinary Research Institute according to the standard protocols and biosafety regulations established by the Institutional Biosafety Committee.

PK-15 cells were seeded on the dishes, and infected with FMDV at an indicated multiplicity of infection (MOI). After incubation for 1 h, the liquid mixed virus was removed and replaced by DMEM containing 2% FBS for indicated times. Subsequently, the cells were collected and analyzed with different experiments.

### 2.3. MiRNA Extraction and Quantification

PK-15 cells were inoculated into 60 mm dishes the night before transfection. When the cells were adhered, the mimics, inhibitor or scrambled negative-control (NC) of miR-4334-5p (GenePharma, Shanghai, China) were transfected with Lipofectamine 2000 reagent (Invitrogen, Carlsbad, CA, USA) according to the manufacturer’s instruction respectively, and the sequences were shown in Table 1. The mature sequence of ssc-miR-4334-5p (Accession: MIMAT0017966) is: 5′-CCCUGGAGUGACGGGGGUG-3′. Cells were challenged with FMDV at 0.1 or 1 MOI, 24 h or 36 h post transfection. MirVana miRNA Isolation kit (Invitrogen, AM1560, Carlsbad, CA, USA) was used following the protocols offered by manufacturer to extract the total microRNA. Reverse transcription was conducted with miScript II RT kit (Qiagen, 218161, Hilden, Germany). MiR-4334-5p expression was quantified by qRT-PCR with miScript SYBR Green PCR Kit (Qiagen, 218073, Hilden, Germany). According to the report of Heidi Schwarzenbach [18], miR-16 is a most used internal control in miRNA researches. In addition, the expression of miR-16 in our studies were stable, so miR-16 was used as an internal control. The forward primers used in qRT-PCR for miR-4334-5p and miR-16 were shown in Table 1; the reverse primer is universal, which was obtained from miScript SYBR Green PCR Kit.

### 2.4. Transfection and Luciferase Reporter Assay

The potential binding sites of miR-4334-5p in ID1 3′-UTR was predicted at position 591~608 by RNA22 (https://cm.jefferson.edu/rna22/) and BiBiServ2 (https://bibiserv.cebitec.uni-bielefeld.de/rnahybrid). The wild-type or mutant sequences of ID1 3′-UTR were cloned into pmir-GLO vector (Promega, Madison, WI, USA). The primers used to amplify ID1 3′UTR were shown in Table 2. The day before transfection, BHK-21 cells were placed in 24-well plates, miR-4334-5p mimics or scramble (NC) and β-gal plasmids were co-transfected with wild-type or mutant ID1 3′-UTR plasmids, respectively, by using Lipofectamine 2000 (Invitrogen, Carlsbad, CA, USA) according to the instructions provided by manufacture. Luciferase activity was determined using Dual Luciferase Reporter Assay System (Promega, Madison, WI, USA) 24 h post transfection, and the results were normalized with β-gal.

### 2.5. Quantitative Real-Time PCR

The cells were harvested at 6 h or 8 h post-infection, and total RNA was isolated with TRIzol (Invitrogen, 15596018, Carlsbad, CA, USA). For reverse transcription of the first strand cDNA, PrimeScript RT reagent Kit with gDNA Eraser (Takara, RR047A, Tokyo, Japan) was used under the instructions of the reagents. The expression of ID1, VP1, and various cytokines was quantified by quantitative real-time PCR (qRT-PCR) using SYBR Premix Ex Taq II (Tli RNaseH Plus) (Takara, RR820A, Tokyo, Japan), and β-actin was set as an internal control. All the primers used for qRT-PCR are shown in Table 3.

### 2.6. Enzyme-Linked Immunosorbent Assay

The mimics, or scrambled negative-control (NC) of miR-4334-5p were transfected into PK-15 cells with Lipofectamine 2000 reagent according to the manufacturer’s instruction, respectively. At 36 h post-transfection, 0.1 MOI FMDV were used to challenge the transfected cells for 6 h. Finally, the supernatant was collected to quantify the expression of IFN-β with IFN-β ELISA Kit, following the manufacturer’s protocols.

### 2.7. Western Blotting

PK-15 cells were inoculated into 60 mm dishes the night before transfection. When the cells were adhered, the mimics, inhibitor or scrambled negative-control (NC) of miR-4334-5p were transfected with Lipofectamine 2000 reagent according to the manufacturer’s instruction, respectively, cells were challenged with FMDV, 24 h or 36 h post transfection. Cells were harvested and lysed with lysis buffer including 1% protease inhibitor (Invitrogen, Carlsbad, CA, USA). After centrifuge, the supernatant was added protein loading buffer and heat-denatured. The samples were subjected to western blot to detect the expression of VP1 and ID1, with indicated primary antibodies, β-actin was set to show the even loading. Visualization of each protein bands were performed with ECL western blot substrate (Invitrogen, Carlsbad, CA, USA) according to the instructions provided by the manufacturer. The relative expression of each protein was quantified by Image J.

### 2.8. Bioinformatics Analysis

TargetScan (http://www.targetscan.org/), PITA (http://genie.weizmanm.ac.il/pubs/mir07/mir 07-.data.html), and miRanda (http://www.microrna.org/microrna/home.do) were used jointly to predict the targets of miR-4334-5p. In order to make an explanation of the possible mechanism of miR-4334-5p in viral propagation, we found out the potential targets and drew networks of microRNA-gene interaction with cytoscape3.4.0 (https://cytoscape.org/).

### 2.9. Statistics Analysis

All of the above experiments were operated at least three times. All data were presented as mean ± S.D. Statistical significance was calculated using one-tailed Student’s *t*-test, and *p* < 0.05 was considered significant (* *p* < 0.05; ** *p* < 0.01; *** *p* < 0.001).

## 3. Results

### 3.1. MiR-4334-5p Expression Was Induced by FMDV

**Figure 1:**
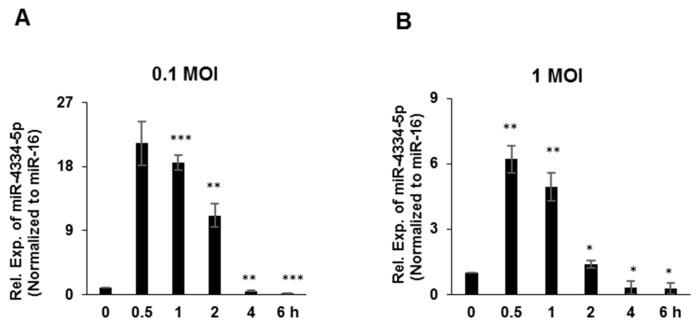
MiR-4334-5p expression was induced by FMDV. (**A**,**B**). Porcine PK-15 cells were challenged with FMDV at 1 MOI and 0.1 MOI. Cells were harvested at indicated time points to examine the expression of miR-4334-5p by qRT-PCR, and miR-16 was detected as an internal control. The data shown represent of three independent experiments with similar results, and normalized to miR-16; Error bars is standard deviation (SD). Significance was calculated by Student’s t-test, *** *p* < 0.001; ** *p* < 0.01; * *p* < 0.05.

In order to validate the previous results of microarray-based miRNAs profiling experiment, PK-15 cells were challenged by FMDV (O/BY/CHA/2010) at 0.1 MOI or 1 MOI, separately. qRT-PCR was applied to quantify miR-4334-5p expression. The expression level of miR-4334-5p was rapidly upregulated at around 0.5 h and continued increasing till 2 h, and following decreased post 4~6 h. Compared with control (0 h), the expression level of miR-4334-5p was more than 21-fold higher in cells infected with 0.1 MOI (Figure 1A) at 0.5 h post infection, and also in 1 MOI infection group it is 6-fold higher compared with control (Figure 1B). After that, the expression level of miR-4334-5p decreased continuously, at 6 h post infection, compared with control (0 h), miR-4334-5p expression was less than 10% in cells infected with 0.1 MOI (Figure 1A), and the level decreased to 15% in 1 MOI infection group (Figure 1B). This sharply and dramatic change of miR-4334-5p during FMDV infection implied it might involve in the regulation on FMDV replication.

### 3.2. FMDV Replication Was Up-Regulated by miR-4334-5p Mimics

**Figure 2:**
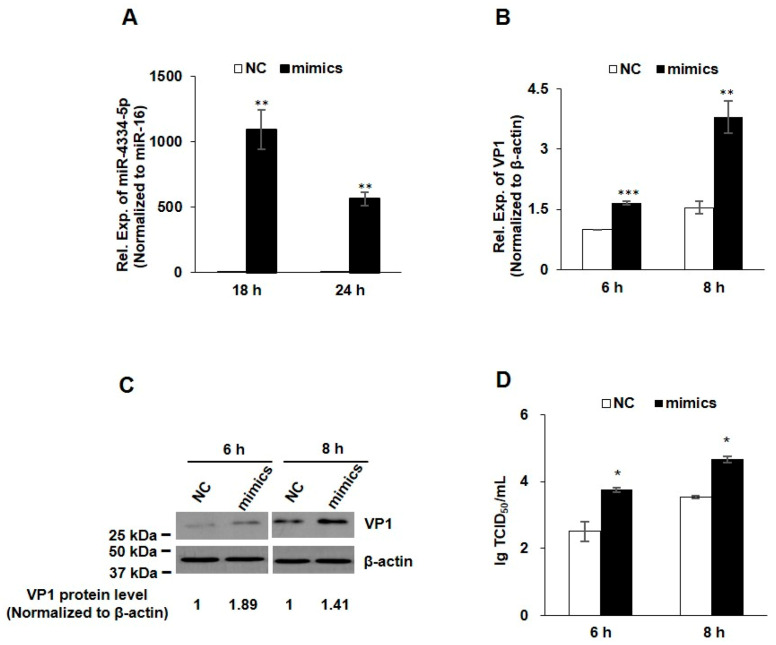
FMDV replication was up-regulated by miR-4334-5p mimics. (**A**). MiR-4334-5p mimics or scramble mimics (NC) were transfected into PK-15 cells for 18 h or 24 h, respectively. Cells were collected and then quantified miR-4334-5p expression by qRT-PCR, and miR-16 was examined as an internal control in parallel. Data shown are means ± SD from triplicate assays, ** *p* < 0.01. (**B**). MiR-4334-5p mimics or scramble mimics (NC) were transfected into PK-15 cells for 24 h, cells were challenged with FMDV at 0.1 MOI for 6 h or 8 h, and then cells were harvested to quantify VP1 expression by qRT-PCR, and the expression level was normalized to β-actin. The data are means ± SD from triplicate assays, *** *p* < 0.001; ** *p* < 0.01 (**C**). PK-15 cells were treated as in (**B**), the cells were lysed and then subjected to Western blot, VP1 and β-actin antibodies were used to detect the protein expression. (**D**). PK-15 cells were treated as in (**B**), after infected with the indicated time, the supernatants were collected to measure the virus titers by TCID_50_ (Median Tissue Culture Infectious Dose) assay. Results shown are means ± SD from triplicate assays, * *p* < 0.05.

In order to evaluate the possible regulatory function of miR-4334-5p on FMDV reproduction, the mimics and scrambled negative-control (NC) RNAs of miR-4334-5p were synthesized and transfected into PK-15 cells. As shown in Figure 2A, compared with NC groups, the miR-4334-5p expression increased more than 1000-fold at 18 h post transfection of miR-4334-5p mimics, and it increased more than 500-fold at 24 h, which clearly demonstrated that the miR-4334-5p mimics work well in PK-15 cells. To explore the role of miR-4334-5p during FMDV infection, the mimics or scrambled negative-control (NC) of miR-4334-5p were transfected into PK-15 cells separately, and then challenged with FMDV at 0.1 MOI post transfection. Compared with in the control cells, transfection of miR-4334-5p mimics promoted FMDV propagation, in Figure 2B–D, the virus structural protein VP1 level (qRT-PCR or Western-blot) and virus titers all increased significantly. All of the above data clearly revealed that the up-regulation of miR-4334-5p promotes FMDV replication.

### 3.3. FMDV Replication Was down-Regulated by miR-4334-5p Inhibitors

**Figure 3:**
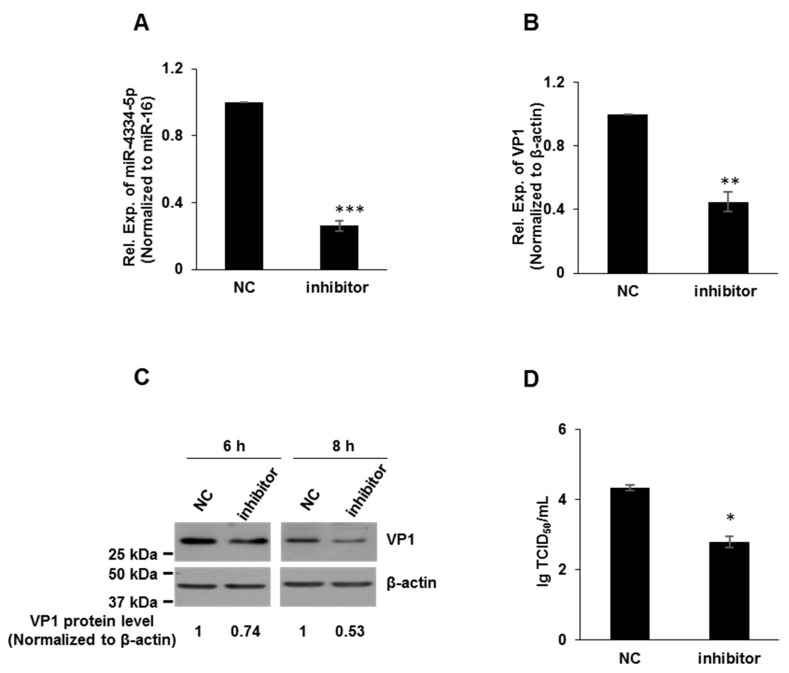
FMDV replication was down-regulated by miR-4334-5p inhibitors. (**A**). MiR-4334-5p inhibitors or scramble control (NC) were transfected into PK-15 cells for 24 h independently. Cells were harvested and quantified miR-4334-5p expression by qRT-PCR, and miR-16 was examined as an internal control in parallel. Data shown are means ± SD from triplicate assays, ***, *p* < 0.001. (**B**). MiR-4334-5p inhibitors or scramble control (NC) were transfected into PK-15 cells for 24 h, cells were challenged with FMDV at 0.1 MOI for 8 h, and then cells were harvested to quantify VP1 expression by qRT-PCR, and the expression level was normalized to β-actin. The data are means ± SD from triplicate assays, **, *p* < 0.01 (**C**). PK-15 cells were treated as in (**B**), the cells were lysed and then subjected to Western blot, VP1 and β-actin antibodies were used to detect the protein expression. (**D**). PK-15 cells were treated as in (**B**), after infected with the indicated time, the supernatants were collected to measure the virus titers by TCID_50_ (Median Tissue Culture Infectious Dose) assay. Results shown are means ± SD from triplicate assays, *, *p* < 0.05.

In order to further evaluate the possible regulatory function of miR-4334-5p on FMDV reproduction, miR-4334-5p inhibitors and scrambled negative-control (NC) RNAs were synthesized. Compared with the transfection of NC, inhibitors repressed the expression level of miR-4334-5p to less than 40%, which demonstrated the knock-down efficiency is significant (Figure 3A). To further validate the role of miR-4334-5p on FMDV reproduction, the inhibitors and scrambled negative-control of miR-4334-5p were transfected into PK-15 cells, respectively, and then challenged with FMDV at 0.1 MOI post transfection. Compared with the control groups, the VP1 expression (qRT-PCR or Western blot) and virus titers all decreased significantly after the transfection of miR-4334-5p inhibitor (Figure 3B–D). In conclusion, all these data clearly suggested FMDV replication was down-regulated by miR-4334-inhibitors.

### 3.4. Bioinformatics Analysis Demonstrated miR-4334-5p Participated in Interferon Regulation

In order to find out the possible targets of miR-4334-5p during FMDV infection, TargetScan, PITA and miRanda were used. There were 5704 targets in total. These proteins were enriched by GO annotation based on three categories: biological process, cellular component, and molecular function. The top enriched terms are concentrated on: single-organism transport, single-organism localization, transport, establishment of localization, single-organism process, localization, regulation of signaling, regulation of cell communication, single-organism cellular process, and single-organism intracellular transport (Supplemental Figure S1A). Protein’s KEGG database description was annotated by using Kyoto Encyclopedia of Genes and Genomes online service tools. KEGG enrichment analysis suggests the target proteins of miR-4334-5p were enriched in cGMP-PKG signaling pathway, PI3K-Akt signaling pathway, amoebiasis, chemokine signaling pathway, estrogen signaling pathway, bacterial invasion of epithelial cells, AMPK signaling pathway, Insulin resistance, pathways in cancer, and Apoptosis-multiple species (Supplemental Figure S1B). The software cytoscape3.4.0 were applied to generate miR-4334-5p-target gene interaction networks. As shown in Supplemental Figure S1C, ID1, IRF4, IRF6, STAT2, STAT3, STAT5A, TRAF1, TRAF6, IL1, IL6 and other virus response genes may be the potential targets of miR-4334-5p.

### 3.5. miR-4334-5p Directly Targets the 3′-UTR of ID1 Gene

**Figure 4:**
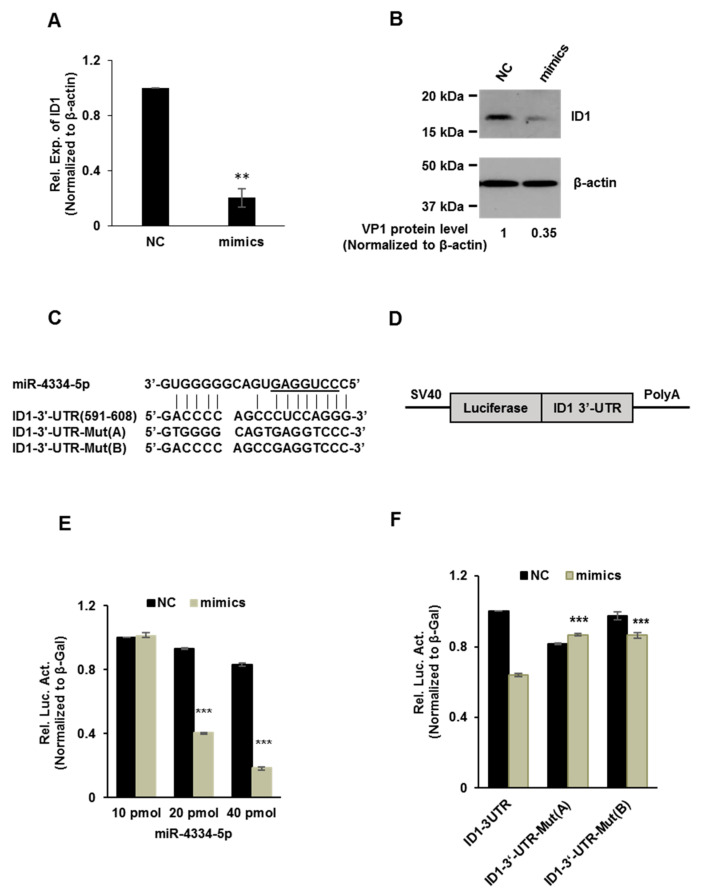
miR-4334-5p directly targets the 3′-UTR of ID1 gene. (**A**). qRT-PCR analysis of the effect of miR-4334-5p on ID1 mRNA expression in PK-15 cells transfected with miR-4334-5p mimics or scramble mimics (NC) at 24 h post transfection, normalized to β-actin. The results shown are means ± SD from triplicate assays, **, *p* < 0.01. (**B**) Western blot analysis of miR-4334-5p on ID1 expression in PK-15 cells transfected with miR-4334-5p mimics or scramble mimics (NC) at 24 h post transfection, and β-actin was used as a control to indicate the even loading (**C**). Bioinformatic prediction of interaction between miR-4334-5p and the 3′-UTR of swine ID1 using RNA22 and BiBiServ2. For each schematic, the upper sequence is mature miR-4335-5p, the middle sequence is the binding site of miR-4334-5p in 3ʹ-UTR of swine ID1, and the lower sequence is the mutated sequence of 3′-UTR in ID1 gene. The seed sequence is underlined. (**D**). Schematic overview of the putative binding sites or mutation of miR-4334-5p binding-sites in 3′-UTR of ID1 mRNA. (**E**). The ID1 luciferase reporter plasmid was co-transfected with various amount miR-4334-5p mimics or scramble mimics (NC) into BHK-21 cells. Data are represented of relative luciferase activities from triplicate assays, ***, *p* < 0.001. (**F**) The specific binding activity of miR-4334-5p mimics or scramble mimics (NC). Data are represented of relative luciferase activities from triplicate assays, ***, *p* < 0.001.

MiRNAs bind on the 3′-UTR of target genes to exert biological functions and repress the translation. In order to validate the above possible targets, we detected the expression of different genes post transfection of miR-4334-5p mimics or scramble negative control (NC), as shown in Figure 4A,B, ID1 mRNA level and protein level were both significantly reduced compared to NC. This suggests that ID1 expression level was impaired by miR-4334-5p. To explore which target sequence could miR-4334-5p bind on, RNA22 and BiBiServ2 were used to predict possible binding targets of miR-4334-5p. The results showed a potential binding site at the position of 591-608 of ID1 3′-UTR, as shown in Figure 4C. To verify the bioinformatic analysis, 3′-UTR containing normal ID1 mRNA or the mutant were cloned into the pmirGLO multiple cloning site (MCS) vector, schematic overview of the putative binding sites or mutation of miR-4334-5p binding-sites in 3′ UTR of ID1 mRNA shown in Figure 4D. These plasmids were co-transfected into BHK-21 cells with miR-4334-5p mimics or scramble negative control (NC) and luciferase activity was determined. As shown in Figure 4E, with an increasing amount of miR-4334-5p mimics, the relative luciferase activity reduced continuously, which clearly showed the suppression of miR-4334-5p on ID1 expression. Furthermore, more interestingly, compared to the normal ID1-3′-UTR plasmid, transfection of ID1-3′-UTR-Mut plasmid, the relative luciferase activity is not regulated by the transfection of miR-4334-5p mimics. These results clearly revealed that ID1 is a direct target of miR-4334-5p.

### 3.6. The Interferon and Antiviral Genes Expression Was Suppressed by miR-4334-5p via Targeting ID1

**Figure 5:**
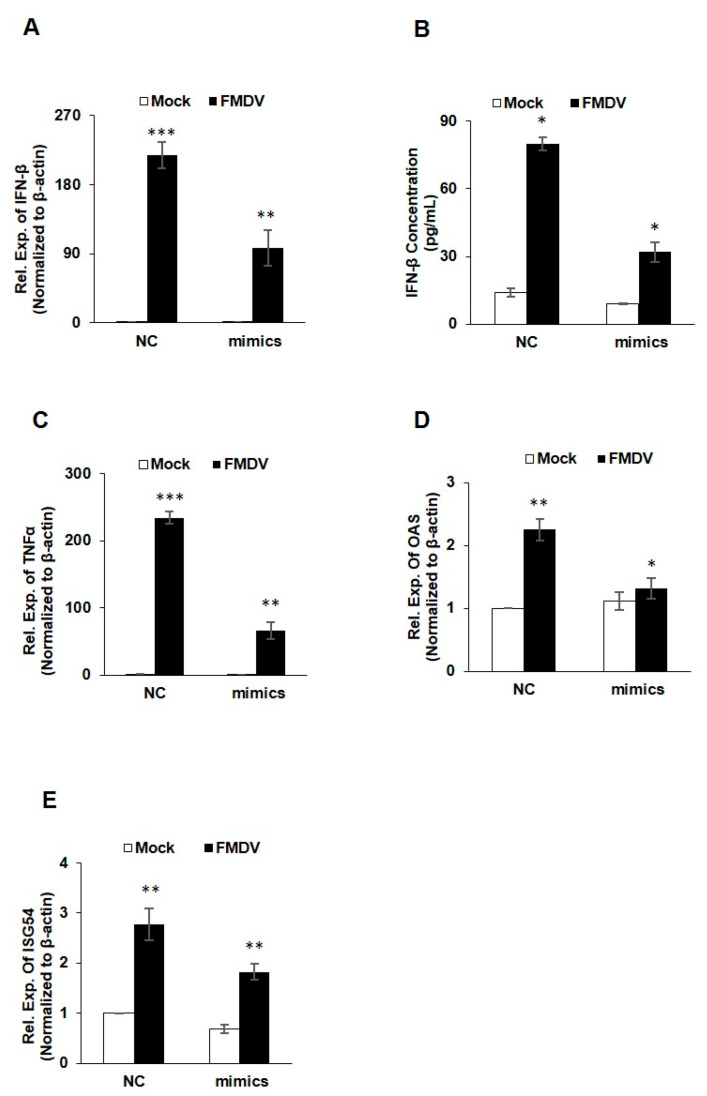
The interferon and antiviral genes expression were suppressed by miR-4334-5p via targeting ID1. (**A**,**B**). MiR-4334-5p mimics or scramble mimics (NC) were transfected into PK-15 cells for 24 h, respectively. Cell were infected with FMDV at 0.1 MOI for another 8 h. And then cells were harvested to detect the IFN-β expression level by qRT-PCR and IFN-β ELISA kit. Results shown are representative of three independent experiments, with each performed in triplicates. Bars represent SD of the mean. Asterisks indicate significance, according to Student’s t-test, ***, *p* < 0.001; **, *p* < 0.01, *, *p* < 0.05. (**C**–**E**). PK-15 cells were treated in the same way as in (**A**,**B**). Cells were harvested to quantify TNFα, OAS and ISG expression by qRT-PCR, β-actin was determined as an internal control. Results shown are means ± SD from triplicate assays. ***, *p* < 0.001; **, *p* < 0.01; *, *p* < 0.05.

Viral RNA acts as a pathogen-associated molecular patterns (PAMPs), which can be recognized by the host pattern-recognition receptors (PRRs) to activate the innate immune signals, and trigger the expression of type-I IFNs and IFN-stimulated genes (ISGs) [19]. In order to validate the above bioinformatics analysis of miR-4334-5p targets, interferon β (IFN-β) expression was quantified by qRT-PCR and ELISA. Compared with the control groups, FMDV infection induced a significant lower IFN-β production transfected with miR-4334-5p mimics, which suggested the miR-4334-5p inhibits interferon production (Figure 5A,B). Based on our previous studies, ID1 was found as a critical antiviral gene. We want to investigate whether the promoted IFN expression was depend on the inhibition of ID1, the IFNs expression were determined by qRT-PCR in peritoneal macrophages and bone marrow macrophages isolated from wild type mice or ID1 knockout mice post FMDV infection, compared with the wild type cells, ID1 knockout cells showed an decreased expression level of IFNs (Submitted to another journal, shown in the supporting figures). These data suggested that miR-4334-5p induced IFNs expression by targeting ID1 during FMDV infection. Some other antiviral genes expression was also quantified by qRT-PCR. Compared with the control groups, TNFα, OAS and ISG54 expression was suppressed after FMDV infection (Figure 5C–E). These results clearly proved that miR-4334-5p repressed interferon production and antiviral genes expression during FMDV infection.

## 4. Discussions

Emerging evidences indicate that the host microRNAs (miRNAs) have a significant effect on the comprehensive host-pathogen interaction [7], even participating in the viral life cycle. For instance, miR-101 represses feline herpesvirus 1 reproduction by targeting cellular suppressor of cytokine signaling 5 to promote IFN-I signaling [20]; miR-7 suppresses rotavirus replication via targeting viral NSP5 directly [21]; miR-122 reduces HBV gene expression and replication via binding with hepatitis B virus pre-genomic RNA sequence [22]; miR-21-3p promotes influenza A virus H5N1 replication by regulating FGF2 to repress IFN-I signaling [23]; miR-545 promotes enterovirus 71 propagation through directly targeting phosphatase and tensin homolog and tumor necrosis factor receptor-associated factor 6 [24]. In the case of FMDV, there are only a few studies, artificial miRNA targeting 3D polymerase is efficient to inhibit FMDV replication [25], miR-1307 and miR-203a repress FMDV infection [2,15]. However, the underlying mechanisms of porcine miR-4334-5p affecting viral replication are still not understood. Here we discovered miR-4334-5p was altered remarkably post FMDV infection (Figure 1). The following experiments clearly showed that overexpression of miR-4334-5p directly accelerated the FMDV reproduction (Figure 2), while the virus replication was decelerated when transfected miR-4334-5p inhibitors (Figure 3). More importantly, luciferase assays demonstrated that miR-4334-5p directly targets the 3′-UTR of ID1 gene (Figure 4), and thus refrains interferon production and antiviral gene expression (Figure 5). In conclusion, these results proved that miR-4334-5p is a novel host miRNA that promotes FMDV replication.

Pattern recognition receptors in the host could recognize viral pathogen-associated molecular pattern, which in turn triggers a series of signaling cascades, including the expression of IFN-I, proinflammatory cytokines (IFN-β and IFN-α, IL-6, IL-1, and TNFα), and chemokines (CXCL10, CCL2, and CXCL8) [3,5]. Proinflammatory cytokines and type I IFNs contribute to the prevention of viral infection [7]. FMDV infection was reported to impair IFN-I expression in PK-15 cells, and correspondingly decrease ISGs (PKR, OAS, and Mx1) to accelerate its replication [4]. In our previous studies, we found ID1 regulate IFN production through FOXO1 (Submitted to another journal). Here we also showed miR-4334-5p directly targets the 3′UTR of ID1 gene (Figure 4), there is only one predicted binding site in the 3′-UTR of ID1 which miR-4334-5p could bind, and even the further investigations suggests that miR-4334-5p overexpression decreased the expression level of IFN-β, ISG54, OAS and TNFα, compared with the scramble groups (NC) (Figure 5). These data demonstrated the significant role of miR-4334-5p on the regulatory of interferon signaling pathways in PK-15 cells together.

Foot-and-mouth disease virus (FMDV) is the pathogen foot-and-mouth disease, which is one of the most contagious viruses. The disease mainly occurred in cloven-hoofed animals, such as cattle, sheep, goats, and swine, clinical symptoms in infected animals usually characterized as the vesicles formation in the mouth, nose, tongue and skin between the claws of the feet [26]. There were multiple artificial designed miRNAs targeting viral 3D^pol^ or internal ribosome entry site, which had been proved to impair FMDV propagation significantly [25]. In addition, miRNA-based treatment of Hepatitis C infection has successfully completed phase II clinical trial [27]. Our data provided a further understanding of cellular miR-4334-5p influencing FMDV replication, which may benefit to the identification of novel therapeutic small RNA molecule drug for anti-viral infection. These researches may promote improvement in exploring a promising noncoding RNA target for diagnosis and treatment of virus infection.

## 5. Conclusions

Here, we showed that miR-4334-5p promotes FMDV replication by interfering with the IFN pathways in porcine cell line PK-15. MiR-4334-5p was significantly increased after FMDV infection. Transfection of the miR-4334-5p mimics promotes FMDV replication, while the results of inhibitor are opposite. We also found miR-4334-5p directly binding to the 3′-UTR of ID1 (Inhibitor of DNA binding 1), which was found to be a critical antiviral factor in our previous studies. These data provide a possible therapeutic pathway to prevent virus replication by targeting miR-4334-5p during FMDV infection.

## Figures and Tables

**Table 1:** Oligos related to the detection of ssc-miR-4334-5p.

Oligo	Sequence (5′-3′)
ssc-miR-4334-5p-mimics-sense	CCCUGGAGUGACGGGGGUG
ssc-miR-4334-5p-mimics-anti-sense	CCCCCGUCACUCCAGGGUU
ssc-miR-4334-5p-scramble-mimics-sense	GGUGGCGCGUCGAGCGUGA
ssc-miR-4334-5p-scramble-mimics-anti-sense	ACGCUCGACGCGCCACCUU
ssc-miR-4334-5p- Inhibitor	CACCCCCGUCACUCCAGGG
ssc-miR-4334-5p- Scramble-Inhibitor	CCACGGUCGACACCGCCUC
ssc-miR-4334-5p-F	CCCTGGAGTGACGGGGGTG
miR-16-F	GCAGTAGCAGCACGTA

**Table 2:** Primers for ID1-3‘-UTR plasmids construction.

Primer		Sequence (5′-3′)
ID1-3′-UTR-Glo primer	GLO-ID1-NheI-sense	CTAGCTAGCAGCGCCGCCCTTGGGGACCTG
	GLO-ID1-SalI-anti-sense	GCGTCGACTACCAACATCTAAGGCATTT
ID1-3′-UTR- mutant primer	Sense	TGGGGGCAGTGAGGTCCCGGCGAGAG
	Anti-sense	GGGACCTCACTGCCCCCACCGCAGGT

**Table 3:** Primers for quantitative real-time PCR.

Primers	Sequence (5′-3′)
FMDV-VP1-F	GACAACACCACCAACCCA
FMDV-VP1-R	CCTTCTGAGCCAGCACTT
sscISG54-F	CTGGCAAAGAGCCCTAAGGA
sscISG54-R	CTCAGAGGGTCAATGGAATTCC
sscTNFα-F	CGTTGTAGCCAATGTCAAAGCC
sscTNFα-R	TGCCCAGATTCAGCAAAGTCCA
sscOAS-F	AAGCATCAGAAGCTTTGCATCTT
sscOAS-R	CAGGCCTGGGTTTCTTGAGTT
sscIFN-β-F	GCTAACAAGTGCATCCTCCAAA
sscIFN-β-R	AGCACATCATAGCTCATGGAAAGA
sscID1-F	GAGTTGGAGCTGAACTCGGAA
sscID1-R	ACACAAGATGCGATCGTCCG
β-actin-F	GCTGGCCGGGACCTGACAGACTACC
β-actin-R	TCTCCAGGGAGGAAGAGGATGCGGC

## Supplementary Materials

The following are available online at https://www.mdpi.com/2073-4425/11/10/1136/s1, Figure S1: Bioinformatics analysis demonstrated miR-4334-5p participated in interferon regulation.
