# Supplementary material for: MiR-4334-5p Facilitates Foot and Mouth Disease Virus Propagation by Suppressing Interferon Pathways via Direct Targeting ID1
Source: Genes (Basel). 2020 Sep 27;11(10):1136. doi: 10.3390/genes11101136 (PMC7601639; doi:10.3390/genes11101136)
Supplement: Supplementary file 1 [file genes-11-01136-s001.pdf]

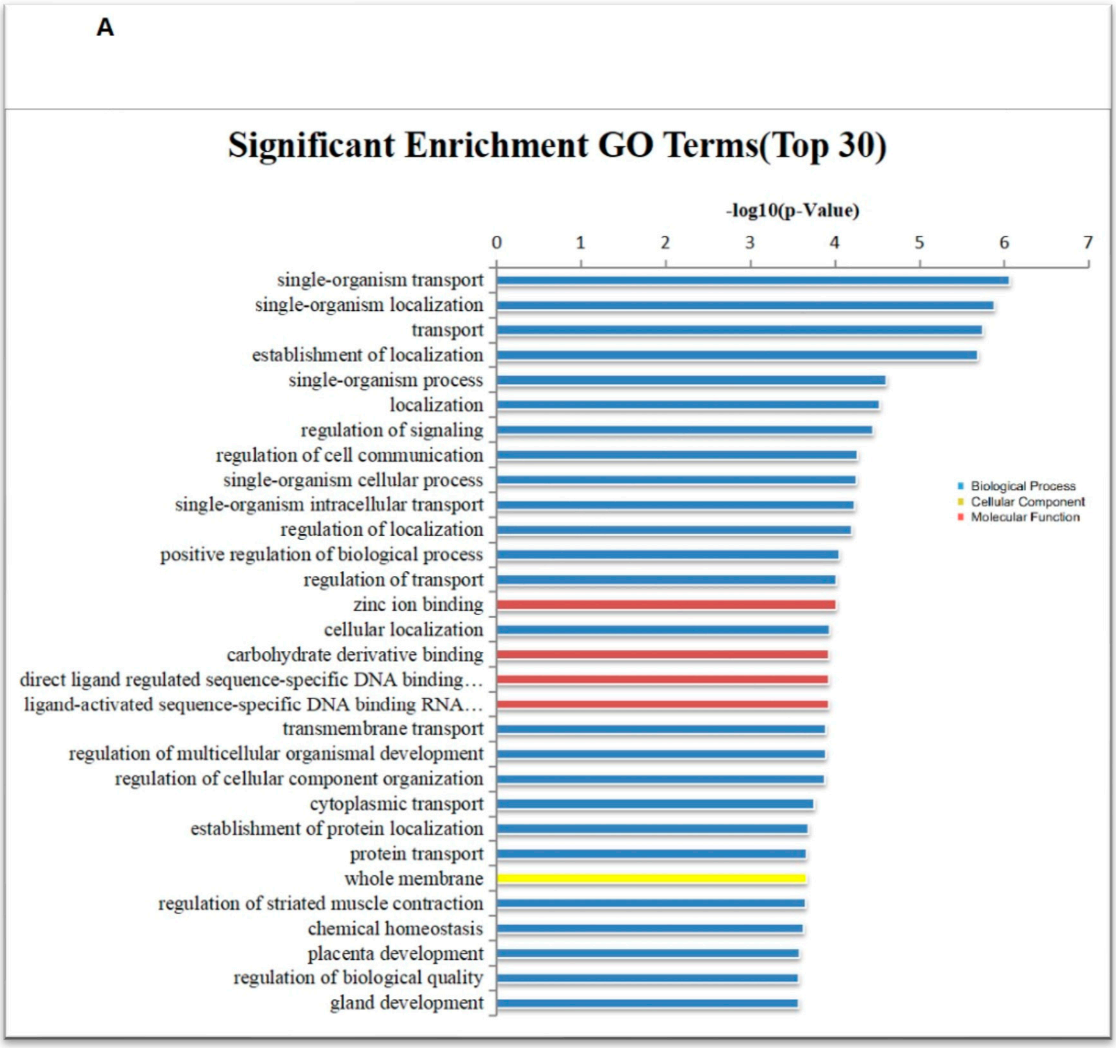

A
Significant Enrichment GO Terms(Top 30)
-log10(p-Value)
0
1
2
3
4
5
6
7
single-organism transport
single-organism localization
transport
establishment of localization
single-organism process
localization
regulation of signaling
regulation of cell communication
single-organism cellular process
single-organism intracellular transport
regulation of localization
positive regulation of biological process
regulation of transport
zinc ion binding
cellular localization
carbohydrate derivative binding
direct ligand regulated sequence-specific DNA binding…
ligand-activated sequence-specific DNA binding RNA…
transmembrane transport
regulation of multicellular organismal development
regulation of cellular component organization
cytoplasmic transport
establishment of protein localization
protein transport
whole membrane
regulation of striated muscle contraction
chemical homeostasis
placenta development
regulation of biological quality
gland development
Biological Process
Cellular Component
Molecular Function

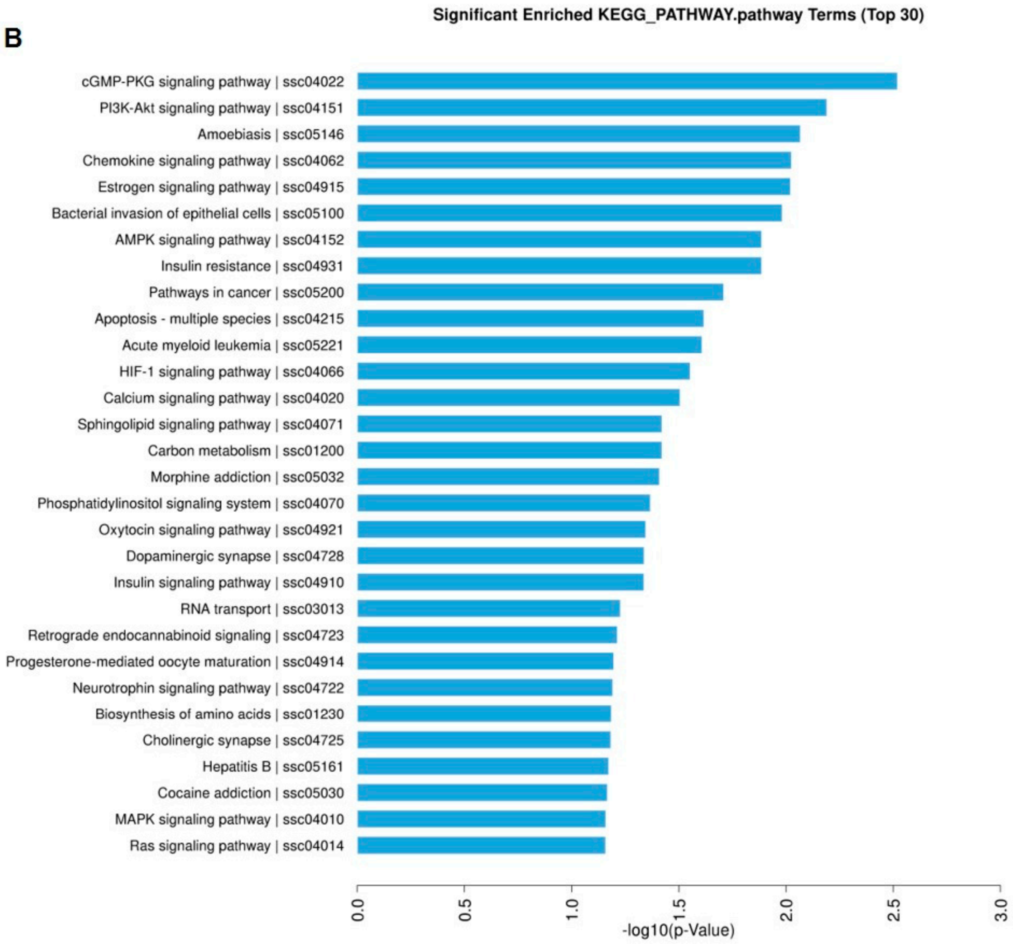

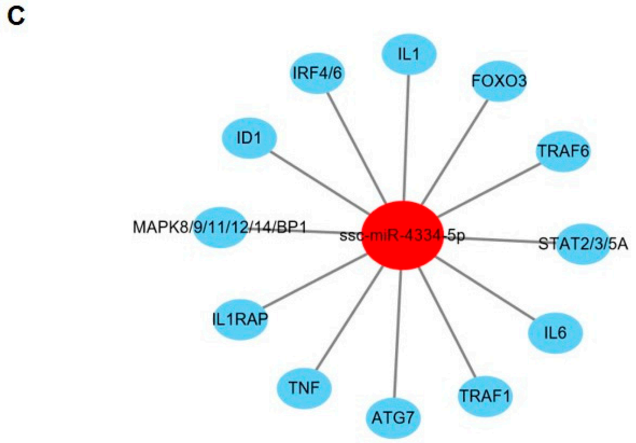

Supplemental Figure 1. Bioinformatics analysis demonstrated miR-4334-5p participated in interferon regulation.

(A). Top 30 significantly enriched gene ontology (GO) terms of miR-4334-5p. The blue bars represent biological process. The yellow bars represent cellular component. The red bars represent molecular function. The ordinate is the GO terms, and the abscissa is the –log (P-value) of GO terms. The length of the bar represents the –log (P-value); credibility increases with bar length. (B). Top 30 significantly enriched pathways of Differentially expressed genes (DEGs) in miR-4334-5p. The ordinate is Kyoto Encyclopedia of Genes and Genomes (KEGG) signaling pathways, and the abscissa is the –log (P-value) of pathways. (C). The target genes

prediction of miR-4334-5p and microRNA-gene interaction networks analysis by software shown in materials and methods. The red dot represents miRNA. Blue dots represent its target genes. Lines represent the regulation relationship of miRNA-target gene.
